# A novel Fe(III) dependent bioflocculant from *Klebsiella oxytoca* GS-4-08: culture conditions optimization and flocculation mechanism

**DOI:** 10.1038/srep34980

**Published:** 2016-10-07

**Authors:** Lei Yu, Qing-wen Tang, Yu-jia Zhang, Rong-ping Chen, Xin Liu, Wei-chuan Qiao, Wen-wei Li, Hong-hua Ruan, Xin Song

**Affiliations:** 1 College of Biology and the Environment, Co-Innovation Center for Sustainable Forestry in Southern China, Nanjing Forestry University, Nanjing, 210037 China; 2 CAS Key Laboratory of Urban Pollutant Conversion, Department of Chemistry, University of Science & Technology of China, Hefei, 230026 China; 3 Key Laboratory of Soil Environment and Pollution Remediation, Institute of Soil Science, Chinese Academy of Science, Nanjing 210008, China

## Abstract

In this work, the effect of cultivation factors on the flocculation efficiency (FE) of bioflocculant P-GS408 from *Klebsiella oxytoca* was optimized by the response surface methodology. The most significant factor, *i.e*. culture time, was determined by gray relational analysis. A total of 240 mg of purified P-GS408 was prepared from 1 liter of culture solution under the optimal conditions. GC-MS analysis results indicated that the polysaccharide of P-GS408 mainly contains Rhamnose and Galactose, and the existence of abundant hydroxyl, carboxyl and amino groups was evidenced by FTIR and XPS analyses. With the aid of Fe^3+^, the FE of kaolin solution by P-GS408 could achieve 99.48% in ten minutes. Functional groups of polysaccharide were involved in the first adsorption step and the zeta potential of kaolin solution changed from −39.0 mV to 43.4 mV in the presence of Fe^3+^ and P-GS408. Three-dimensional excitation-emission (EEM) fluorescence spectra demonstrates that the trivalent Fe^3+^ and Al^3+^ can bind efficiently with P-GS408, while those univalent and divalent cations cannot. With the help of SEM images, FTIR, zeta potential and EEM spectra, we proposed the P-GS408 flocculation mechanism, which consists of coordination bond combination, charge neutrality, adsorption and bridging, and net catching.

Bioflocculants are flocculating agents secreted by microorganisms during their growth and lysis. Due to their good biodegradability and no toxicity, they are widely considered as environmentally friendly compared to chemically synthetic flocculants^1^. It is generally acknowledged that bioflocculant formation involves a very complex process and is affected by many environmental factors. For example, the initial pH of the culture medium may change the electric charge of the cells and redox potential, and further affect the uptakes of nutrients and the rates of enzymatic reaction^2^. In general, a neutral pH is favored for bioflocculant production by many microorganisms^2^,^3^,^4^. Carbon source, nitrogen source and C/N ratio were also reported as key factors affecting bioflocculant production, but the significance of these factors varies for different microbial species^4^,^5^. Moreover, culture time plays a key role on flocculation efficiency (FE) because some strains *e*.*g. Enterobacter cloacae* and *Pseudomonas alcaligenes* would start to degrade the extracellular biopolymer substance (EPS) after depletion of exogenous carbon source^6^. While previous studies were mainly focused on single factor optimization^1^,^5^,^7^, multiple factors optimization for FE was scarcely reported, which is a key for maximizing the yield and cost-controlling.

Response surface methodology (RSM) provides an effective tool for building a multivariable equation and evaluating the optimal values of dependent variable based on statistical principles^8^. Moreover, it is essential to know the most significant independent variables (influential parameter) effect on the yield and cost of bioflocculant production. Grey relational analysis (GRA), one of the most important contents of grey theory, has been employed to evaluate the significance of the influencing factors to many complex processes, *e*.*g*. biohydrogen production, soluble microbial products (SMPs) production and EPS extraction process^9^,^10^. Similar to SMPs, bioflocculant production system can also be considered as grey systems because of its high complexity and lacking of precise information.

To obtain the highest FE, cations are often added: (1) to decrease the negative charge of biopolymers and the suspended particles, and (2) to interact with the carboxyl groups of bioflocculants to form an ionic bonding which stimulates the connection with suspended particles^7^,^11^. Addition of various cations can lead to different FEs of bioflocculation. For example, the FE of bioflocculant CBF-F26 could be enhanced greatly by bivalent and trivalent cations, except Mg^2+ ^12; the FE of bioflocculant excreted from *Aspergillus flavcus* was stimulated by Na^+^, K^+^, Ca^2+^, Mg^2+^ and Mn^2+^, but inhibited by Fe^3+ ^3; while on the other hand, the FE of bioflocculant extracted from *Nannocystis* sp. NU-2 strongly depended on Fe^3+ ^13. Analytical methods, such as SEM, FTIR and Zeta potential analysis have been made to elucidate the stimulation effects on flocculation process by various cations^1^,^13^. However, to the best of our knowledge, the specific stimulation mechanism in presence of various cations is still unclear, especially for the influences of various metals on flocculation efficiency.

Therefore, this work aims to identify the optimization culture conditions for FE of bioflocculant harvested from a model strain, *Klebsiella oxytoca* GS-4-08, which showed great potentials in bioremediation in our previous works^14^,^15^. With the aid of three-dimensional excitation-emission (EEM) fluorescence spectra, the flocculation mechanism, especially the interaction mechanism between the bioflocculant and cations *e*.*g*. Ca^2+^, Al^3+^ and Fe^3+^ has been explored.

## Results

### Optimization of the culture conditions

**Figure 1:**
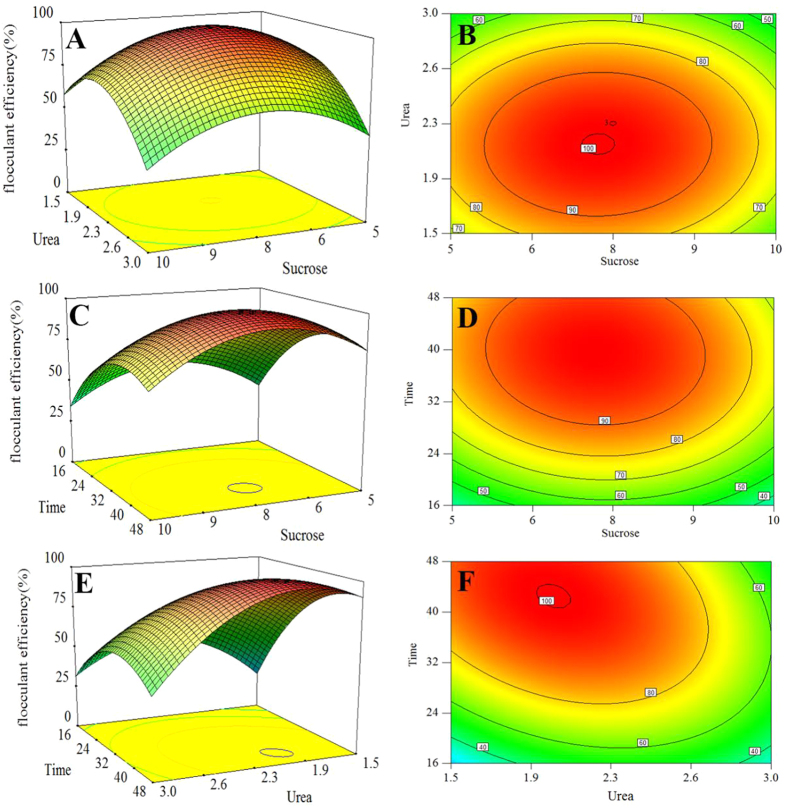
3D surface graphs (A,C,E) and contour plots (B,D,F) of flocculation efficiencies showing the effect of variables: (A,B) Urea- Sucrose; (C,D) Time- Sucrose; (E,F) Time- Urea.

The culture conditions with the corresponding FEs are shown in Table 1. The statistical analysis of the regression model was performed using the Fisher’s statistical method. The interaction between the factors and FEs can be described as Eq. (1):
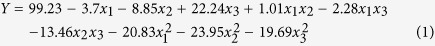
where *x*_1_, *x*_2_ and *x*_3_ are sucrose, urea and culture time, respectively. As shown in Table S1 in Supporting Information, the regression coefficient (*R*^2^) was 0.840, suggesting the regression model was appropriate for simulating the experimental data^16^. Moreover, the value of F-statistic (4.09) was greater than the tabular F_0.05,9,7_(3.68), which indicates the model was significant at a 95% confidence level. As shown in Fig. 1, surface and contour plots of the quadratic model with the FE as the response were constructed. The obvious peaks in the response surfaces and contour plots indicate that the optimal conditions were exactly located within the design boundary. In other words, there were significant interactive effects on FE between sucrose and urea, sucrose and culture time, as well as urea and culture time^17^. By using the “Numerical optimization” tool, a maximum FE (99.49%) was estimated under the optimal culture conditions with sucrose at 5.92 g l^−1^, urea at 2.13 g l^−1^, and a culture time of 36 h. Two confirmation experiments were performed to evaluate the accuracy of the model and the FEs of 99.5% and 99.2% were close to the estimated value further indicating RSM with central composite design (CCD) analysis could be used to describe the relationship between the FE and various factors.

### Influential priority

GRA method was used to evaluate the influential degrees of sucrose concentration, nitrogen concentration and culture time on the FE. The grey relational grade *γ* of these factors for the flocculent efficiency were calculated as:
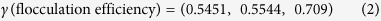
According to the GRA result (Table 1), all the factors have significant effects on the FE, and the culture time shows the most significant effect, followed by urea concentration and glucose concentration.

### Composition analysis

**Figure 2:**
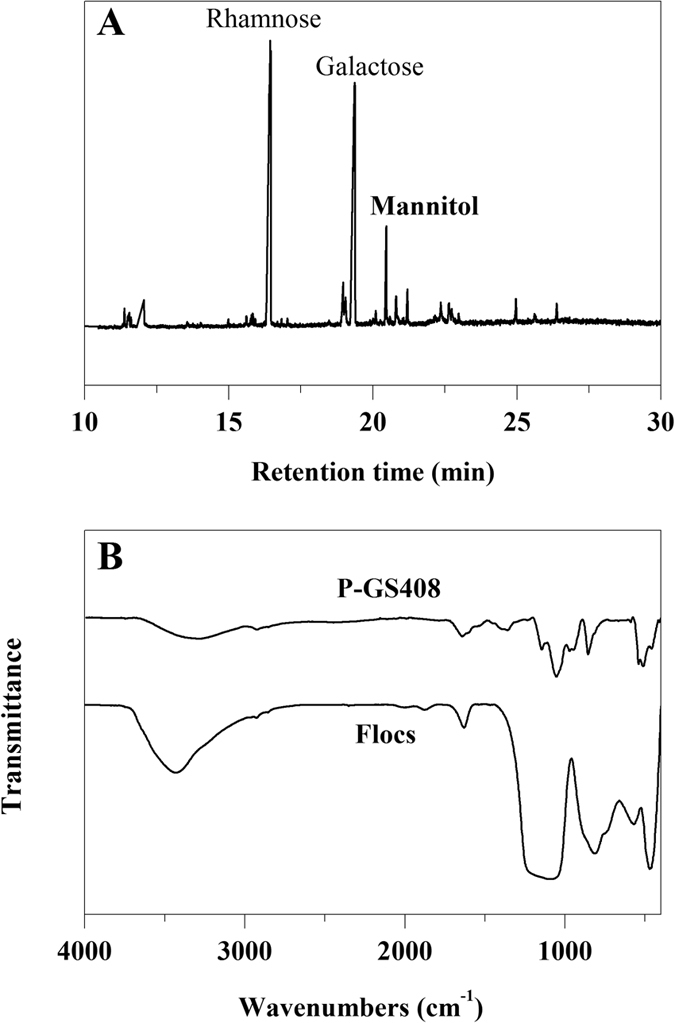
(**A**) Gas chromatogram of alditol acetate derivatives from P-GS408 and (**B**) FTIR spectra of P-GS408 and flocs.

A total of 240 mg of purified P-GS408 was recovered from 1 L of fermentation broth under the optimal condition. The result of color reaction showed that the Anthrone reaction was positive and the results of UV spectrophotometry showed that there were no obvious absorption at 280 nm and 260 nm, confirming that the bioflocculant mainly consisted of polysaccharide with little nucleic acids and proteins (Fig. S1, in Supporting Information). As shown in Fig. 2A, two major carbohydrate peaks at 16.43 and 19.36 min were shown on gas chromatogram, which were attributed to Rhamnose (Rha) and Galactose (Gal) respectively. By comparing the MS data of standard sugars (data not shown), the existence of Rha and Gal in P-GS408 was further confirmed. Moreover, an acetylpyridinium chloride polysaccharide precipitation was formed in mixture of the CPC and P-GS408 solutions, which indicates that P-GS408 contains acidic groups (COO^−^)^6^.

### FTIR and XPS analysis

**Figure 3:**
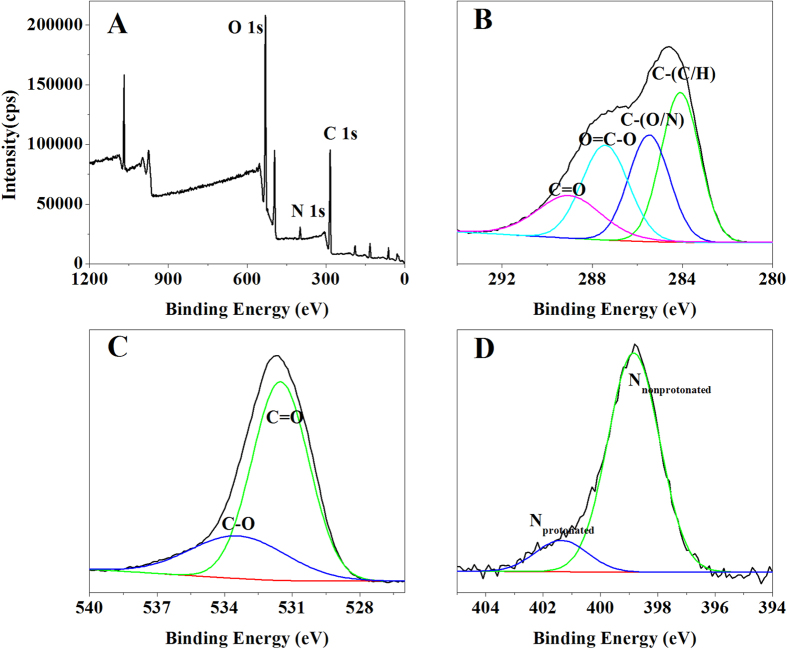
XPS spectra of P-GS408 (A) and high resolution 1 s XPS spectra of C, O, and N from P-GS408 are shown in (B–D), respectively.

As shown in Fig. 2, the broad absorption peak at 3292 cm^−1^ (characteristic of a hydroxyl group) could be attributed to the vibration of −OH or −NH in the sugar ring of polysaccharides^18^. The peak at 2923 cm^−1^ is typical of carbohydrates, an indication of C-H asymmetric stretching vibration. The amide I band at 1643 cm^−1^ is predominantly assigned to C=O stretching associated with aminosugars^19^. The band at 1360 cm^−1^ is attributed to C=O symmetric stretching of carboxyl groups^20^. The peaks at 1143 cm^−1^ and 1053 cm^−1^ are due to sugar derivatives^21^. The sharp absorption peak at 856 cm^−1^ could be attributed to *β*-glycosidic links between sugar monomers. As shown in Fig. 3A, XPS indicates that the core level peaks were C 1s (284.6 eV), O 1s (531.6 eV) and N 1s (398.8 eV), respectively. The analysis results of C 1s, O 1s and N 1s core-level spectra were summarized in Table 2. These results were in agreement with the FTIR analysis and provided further evidence that the hydroxyl, carboxyl and amino groups were abundant in the P-GS408.

### SEM observations

SEM observation was carried out to elucidate the surface morphology of the P-GS408 and its flocculation to kaolin solution. SEM images indicate that P-GS408 had an irregular, sponge structure with many macropores on the coarse surface (Fig. S2A,B in Supporting Information), which was similar to the amorphous structure of a bioflocculant CBF-F26 preparing from mixed culture of *Rhizobium radiobacter* F2 and *Bacillus sphaeicus* F6^12^, but different from the interleaving linear chain-like structure of XMMBF from *Bacillus licheniformis*^11^. Compared to the surface images of P-GS408 (Fig. S2A,B) and kaolin particles (Fig. S3C,D), we can easily find from Fig. S2E,F that P-GS408 connects scattered kaolin particles together, indicating the excellent flocculation ability of P-GS408 to kaolin solution.

### Effect of pH on FE

**Figure 4:**
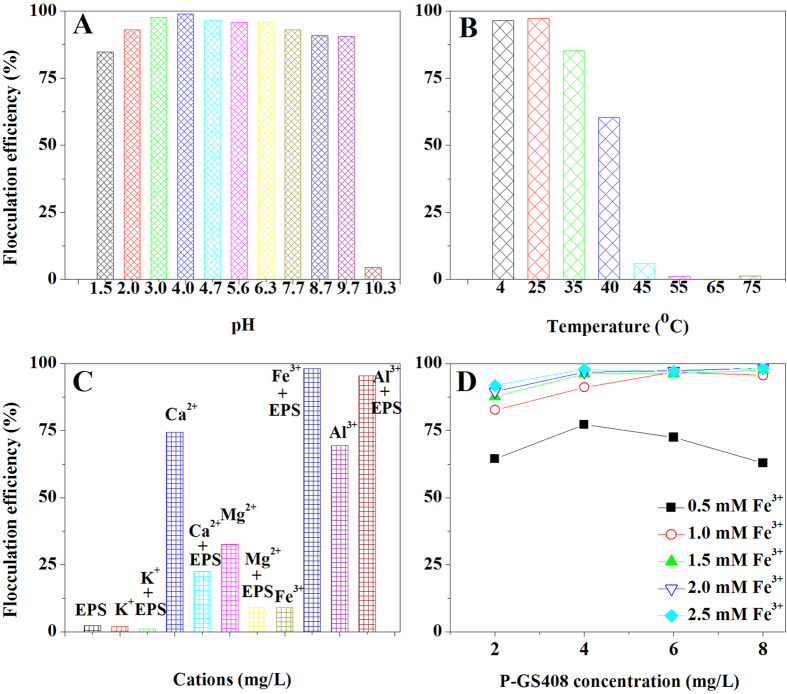
Effects of (**A**) pH and (**B**) temperature on flocculation efficiency (FE). The final concentrations of Fe^3+^ and P-GS408 in test solution were 1.5 mM and 4 mg/L, respectively. (**C**) Effect of various cations on FE. (**D**) Effect of Fe^3+^ concentration on FE with P-GS408 concentration varying from 2 mg/L to 8 mg/L. The final concentrations of cations and P-GS408 were kept at 1.5 mM and 4 mg/L, respectively. All the tests were performed in duplicate, and the results were averaged.

As shown in Fig. 4A 4 mg/L of the P-GS408 demonstrates a high flocculation activity toward kaolin suspension in presence of 1.5 mM Fe^3+^. The P-GS408 showed high FEs within a wide pH range (Fig. 4A), suggesting that it is tolerant to acidic (pH 2.0) and basic (pH 9.7) conditions. It is worth noting that the FE remained at 84.8% even when pH drop to as low as 1.5. This result indicates a much higher acidic tolerance of the P-GS408 than many reported bioflocculants, whose pH optimum usually ranged between 3–9^22^. However, the higher pH value resulted in a lower FE, which may be due to the hydroxide ion (OH-) interfered with the complex formation of the polysaccharide and kaolin particles mediated by Fe^3+ ^6.

### Effect of temperature on FE

The optimum temperature for flocculation was 25 °C (Fig. 4B), similar to the acidic polysaccharide produced by *Enterobacter* sp. BY-29. While at higher temperature, the FE decreased gradually. Some studies reported that FE was decreased at higher temperature due to the breaking down of the polysaccharide chain and an increase in hot movement of kaolin particles which led to the low potential to form bridges with the kaolin particles^6^,^21^. In order to evaluate whether the activate structure of P-GS408 changed after hot treatment, additional thermal stability experiments were performed. The FE maintained at 89.23% after the P-GS408 was exposed at 100 °C for 30 min, indicating that P-GS408 is highly thermostable and the structure cannot be easily changed. This extreme thermos-stability of P-GS408 was better than those of polysaccharides bioflocculant preparing from *Klebsiella* sp. TG-1, *Bacillus mucilaginosus*, *Enterobater cloacae* WD7 and etc.^6^,^7^,^23^.

### Effect of cations on FE

As shown from Fig. 4C, the flocculation was strongly dependent on cations and nearly no flocculation occurred when the P-GS408 was present alone. It was observed that the FEs of P-GS408 with K^+^, Ca^2+^ and Mg^2+^ were lower than in the presence of these cations alone, but were significantly stimulated by Fe^3+^ and Al^3+^. Considering the neurotoxicity of Al^3+^, Fe^3+^ was chosen as the optimal cation with P-GS408 for flocculation. The Fe^3+^ dependent flocculation ability of EPS from *Klebsiella oxytoca* GS-4-08 was similar to some reported strains, *e*.*g*., *Nannocystis* sp. NU-2, *Enterobacter cloacae* WD7^6^,^13^, but different from other strains, *e*.*g*., *Bacillus* sp. F19, *Klebsiella pneumonia* and *Klebsiella* sp. ZZ-3^21^,^22^,^24^.

### Effects of dosage of Fe^3+^ and bioflocculant on FE

Fe^3+^ dosage of 0.5, 1.0, 1.5, 2.0 and 2.5 mM (final concentration) were used to test the FEs in presence of P-GS408, with the concentration varying from 2 to 8 mg/L. As shown from Fig. 4D, all the FEs were below 75% in the presence of 0.5 mM Fe^3+^, indicating that lower concentration of Fe^3+^ can only partially decrease the negative charge on both the biopolymers and particles^22^. Increasing the Fe^3+^ concentration to 1.0 mM or above, the FEs were significantly improved. The FEs varied from 64.4% to 91.8% at the dosage of P-GS408 at 2 mg/L, and the efficiencies were increased correspondingly when the dosage increased to 4 mg/L. This indicates that, at the lower dosage (2 mg/L) of P-GS408, there was insufficient amount of the bioflocculant to be adsorbed on the suspended kaolin particles. Hence, the optimal dosage of P-GS408 and Fe^3+^ were determined to be as 4 mg/L and 1.5 mM for flocculation of kaolin solution in this study.

### Interactions between the P-GS408 and metal cations

**Figure 5:**
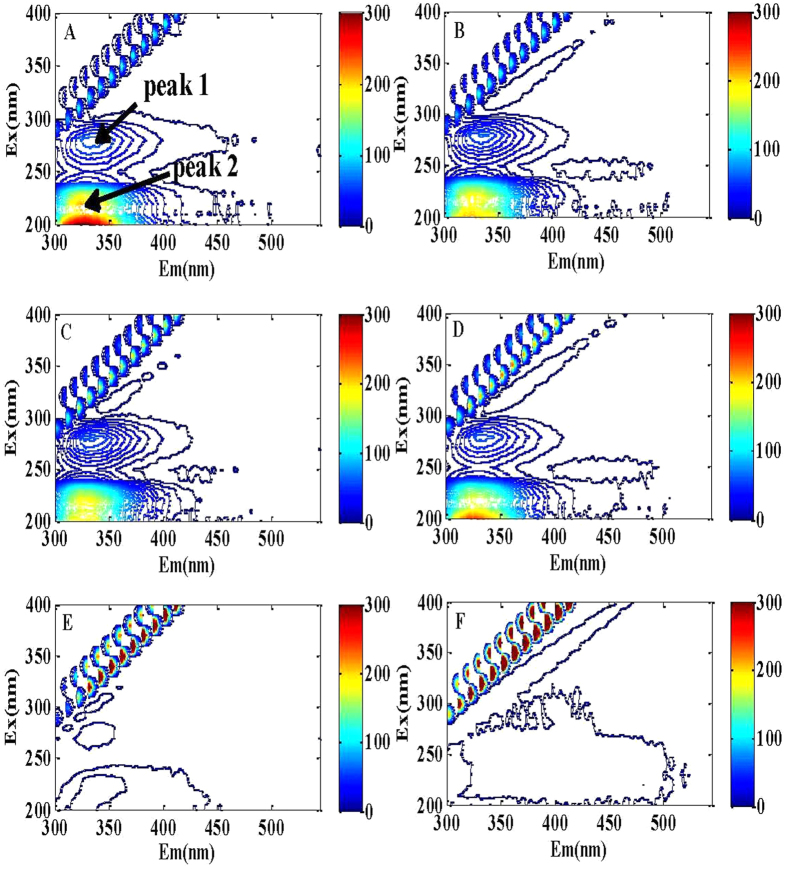
EEM fluorescence spectra of P-GS408 with different metal cations: (A) without any metal; (B) K^+^; (C) Ca^2+^; (D) Mg^2+^; (E) Al^3+^; (F) Fe^3+^. The final concentrations of P-GS408 and metals in analytical solution were 50 mg C/L and 1.5 mM, respectively, except the Fe^3+^ concentration was 1.0 mM.

As shown in Fig. 5A, two fluorescence peaks (Peak A and Peak B) were identified in the EEM spectra of P-GS408. No humic acid-like fluorescence was observed in this study, which is due to the small molecular size of humic acid that would easily vanish after dialysis during the purification step^25^. The first main peak was identified at Excitation/Emission wavelengths (Ex/Em) of 270-280/325-345 nm (Peak 1), and the second main peak was observed at Ex/Em of 210-220/320-330 nm (Peak 2). The two peaks are ascribed to protein-like substances, specifically the aromatic amino acid tryptophan^26^.

**Figure 6:**
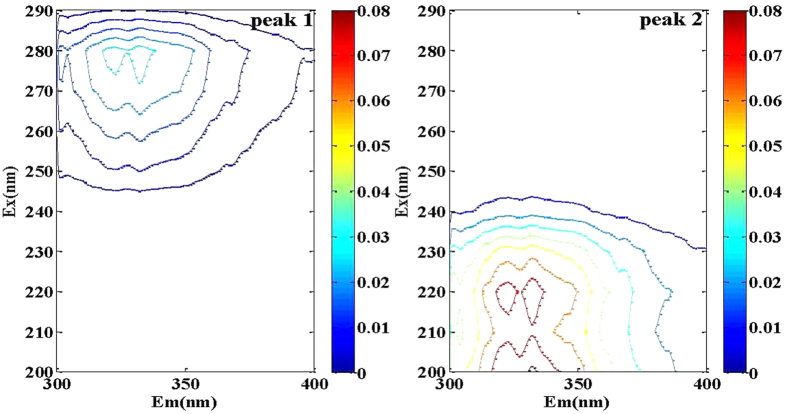
EEM spectra of two main fluorescence components obtained from PARAFAC analysis.

It was found that EEM fluorescence spectra of P-GS408 was influenced by K^+^, Ca^2+^ and Mg^2+^ slightly, but by Al^3+^ and Fe^3+^ significantly, especially by Fe^3+^. A dosage of 0.2 mM of Fe^3+^ (much lower than the dosage used in flocculation experiments) produces significant changes on both peaks of EEM spectra. The peak values were negative when the analytical condition was the same as the flocculation condition, indicating a strong binding of Fe^3+^ onto P-GS408 (see in Fig. 3D in Supporting Information). The fluorescence intensity decreased with the increasing Fe^3+^ concentration (Fig. 6C). The parallel factor (PARAFAC) analysis further confirms that P-GS408 contains two main components, and the peak intensities of which were found to decrease with the increasing Fe^3+^ concentration (Fig. 6).

## Discussion

In this work, a novel Fe(III) dependent highly-efficient bioflocculant, P-GS408, was obtained, and the culture conditions for FEs were optimized as well. The carbon and nitrogen preference of this strain is similar with many other isolates^1^,^7^,^27^. With the aid of GRA, the priority influential factor on flocculent efficiency was culture time, followed by urea and sucrose concentration. Culture time not only plays an important role on FE, but also on cost-control. For this strain, the optimal culture time of P-GS408 production was determined as 36 h, which is less than production periods of other bioflocculants^3^,^28^. The less consumed time gives a shortened production period, which is beneficial for achieving a higher yield. Moreover, the cheap nutrition and less culture time obtained in this work indicated that the strain GS-4-08 has a great potential of bioflocculant production in a large scale.

### Flocculation mechanism

It appears the flocculation mechanism of P-GS408 is very complex. No flocculation happened in the absence of Fe^3+^, which may be due to the coordination formation between the Fe^3+^ and COO^−^ in uronic acid. The infrared spectrum of flocs showed the peaks intensity in range of 1100 to 1250 cm^−1^ shifted and/or changed dramatically (Fig. 2B), which further indicates that the functional groups of polysaccharide were involved in the adsorption. Moreover, both the P-GS408 (−30.2 mV) and kaolin surface (−39.0 mV) were negatively charged in neutral environment. But the zeta potentials of P-GS408/kaolin and kaolin/FeCl_3_/P-GS408 solution were −39.7 and 43.4 mV respectively, suggesting that charge neutralization is one of the mechanisms for the flocculation of the P-GS408^29^. Thus, addition of a cation to the flcocculation system was necessary to induce the effective flocculation in this study. But this cannot explain the role of Ca^2+^ and Mg^2+^ in flocculation, which usually increase the initial adsorption of biopolymers on suspended particles by decreasing the negative charge on both biopolymers and particles and finally improved flocculation^11^,^12^.

In this study, the EEM analysis gives direct evidence that none of the cations except for Fe^3+^ could bind efficiently with P-GS408 (Fig. 5). The fluorescence intensity did not change with K^+^, Ca^2+^ and Mg^2+^, indicating that the binding strength between the P-GS408 and those univalent and divalent cations was weak. The occurrence of electrostatic interactions between the cations was evidenced by the increasing zeta potentials (data not shown), but the binding was attributed to charge neutrality of the negatively charged functional groups *e*.*g.*, carboxyl. In other words, the charge neutralization may be involved in the flocculation process but it was not the main reason for the efficient flocculation. In the flocculation system with addition of Fe^3+^, the EEM spectra of P-GS408 was significantly influenced. This result implies that Al^3+^ and Fe^3+^ would combine with the functional groups of P-GS408, resulting in a change in structure and the formation of a stable non-fluorescence complex between P-GS408 and trivalent cations. This was similar to the interaction mechanism between the EPS and heavy metals e.g., Hg^2+^, Cu^2+^ in some previous studies^25^,^30^,^31^.

**Figure 7:**
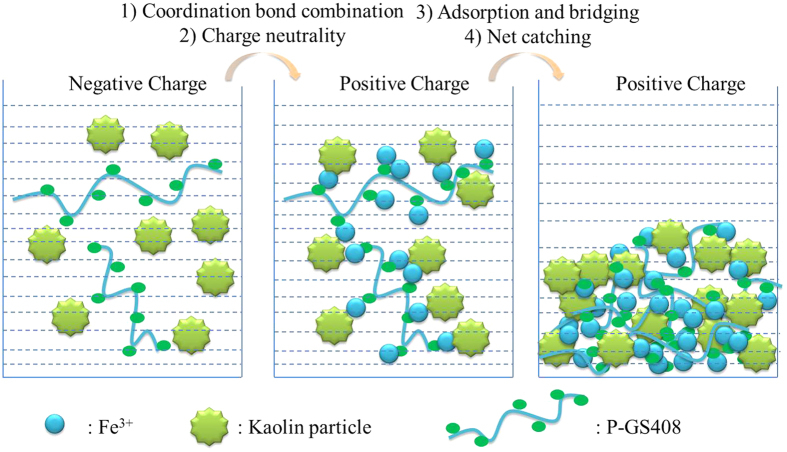
Working model of kaolin particle-Fe^3+^-P-GS408 bioflocculant.

Hereby, we proposed a hypothesis of flocculation mechanism of kaolin suspension by P-GS408 (Fig. 7), which consists of coordination bond combination, charge neutrality, adsorption and bridging and net catching. To start off, Fe^3+^ incorporates with the carboxyl groups of P-GS408, evidenced by FTIR showed in Fig. 2B. After all the binding sites in P-GS408 were saturated with Fe^3+^ (evidenced by EEM spectra showed in Figs 3 and 5), P-GS408 might bind Fe^3+^ through electrostatic interactions and would lead to the charge reversal of P-GS408, as evidenced by zeta potential analysis. Then, the kaolin particles can effectively connect with the P-GS408 through adsorption and bridging. During the settlement process, kaolin particles were gathered and net-caught, evidenced by SEM observations (Fig. S2).

### Significance of this work

Environmental factors such as carbon source, nitrogen source and C/N ratio^4^, and cations were found to crucially affect the FE, while multiple factors optimization for FE was scarcely investigated. Moreover, the roles of cations in flocculation were not clear and inconsistent by conventional analysis, such as SEM, zeta potential and FTIR. In present work, based on the statistical analyses, *i*.*e*., RSM and GRA, the optimal culture condition and influential degrees could be effectively evaluated. Although some factors were negligible in preparation of P-GS408, it is still helpful to maximize the yield and cost-controlling. Furthermore, the interactions between the binding sites of P-GS408 and cations were cleared by EEM analysis, which may beneficial for understanding the flocculation processes and mechanisms when in presence of various cations. Since the FE is highly dependent on the binding of bioflocculants and cations, the approach proposed in this work might also be used to evaluate the effects of cations on widely used flocculants and to explore the flocculation mechanisms.

## Materials and Methods

### Microorganism and bioflocculant extraction

The bacteria *Klebsiella oxytoca* GS-4-08 (CMCC NO. 5237), deposited in the China General Microbiological Culture Collection Center^32^, was used as a model strain to explore the flocculation activity of EPS. The strain was maintained on LB agar medium containing (in g/L): peptone (OXOID) 10, yeast extract (OXOID) 5, NaCl 10 and agar 10. Basal medium was used for microbial growth and bioflocculant production containing following components (in g/L): sucrose 5.92, urea 2.13, KH_2_PO_4_ 2, K_2_HPO_4_ 5, MgSO_4_·7H_2_O 0.5, FeSO_4_·7H_2_O 0.01, and NaCl 0.05. Strain GS-4-08 was cultured aerobically at 35 °C for 36 h.

The preparation and purification method of bioflocculant was performed according to Yuan, *et al*.^20^. The pH of culture broth was adjusted to 12.5 with 0.2 M of NaOH solution, and EPS was removed from cell surface by sonication for 10 min. After refrigerated centrifugation at 8,000 × *g* for 15 min, the supernatant liquid was collected. Cold ethanol was added to the supernatant and this was followed by settling overnight at 4 °C. The white precipitate was collected and dissolved in deionized water and then dialyzed (molecular weight: 14000, Biosharp, USA) against deionized water. Finally, the liquid in dialysis bag was lyophilized to obtain the crude EPS, which designed as P-GS408.

### Measurement of FE

The flocculation activity of the EPS was evaluated by measuring the FE toward kaolin (6000 mesh, 3.5 μm, Aladdin) suspension as model system in a 100 mL beaker. Kaolin suspension of 50 mL (3.5 μm, 4 g/L) was mixed with 200 μl of EPS (1 g/L) and 150 μl of FeCl_3_(1 M). The mixture was fast stirred for 30 s (300 rpm) and low stirred (50 rpm) for 30 s and then settled for 10 min. 1.5 mL of supernatant was carefully removed and afterward the absorbance was measured at 550 nm (A). A control experiment was performed by adding the same volume of deionized water instead of EPS, and carried out in the same way to get absorbance at 550 nm (B). All batch tests were conducted in duplicate. The FE of EPS was calculated as follows
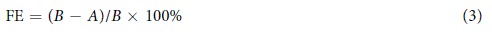


### Optimization of the cultivation conditions for flocculation

A three factor-tree coded CCD of experiments and RSM were applied to optimize the three key variables: glucose dosage, nitrogen dosage and incubation time. The experimental design table for P-GS408 production is shown in Table 1. The response variable (*Y*) that represented flocculation activity was fitted by a second-order model in the form of quadratic polynomial equation:
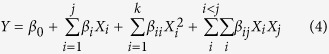
where *Y* is the predicted response, *X*_*i*_ and *X*_*j*_ are independent factors, *β*_*0*_ is the intercept, *β*_*i*_ is the linear coefficient, *β*_*ii*_ is the quadratic coefficient, and *β*_*ij*_ is the interaction coefficient. Design Expert software (version 8.0) was used for the regression and graphical analysis of the data. Finally, two additional experiments were conducted to verify the validity of the statistical experimental strategies.

### Grey relational analysis

To evaluate the importance of various factors on the FE, independent input variables (glucose dosage, nitrogen dosage and incubation time) and the output variable (FE) was composed an *n* × *m* matrix *X*, where *m* is the total number of factors to be considered and *n* is the total number of observation data. The *m* and *n* value were determined as 3 and 17, respectively, according to the RSM design table in this study.

It is necessary to normalize the original data prior to GRA. The normalized data *X*_*i*_(*k*) were calculated with the extended method of mean value as follows^10^:
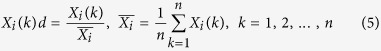
where 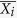 is the average value of *X*_i_(*k*), *X*_i_(*k*)*d* is the value of *X*_i_(*k*) relative to 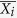. The grey relational coefficients, *ξ*_*i*_ *(k)*, were used to express the relationship between the factors and the flocculating efficiencies which could be expressed as equation (6):
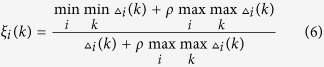
where, 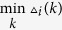 is the element of minimum value in the matrix *∆* and 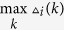 is defined as the element of maximum value in the matrix *∆*, *ρ* (0 < *ρ* < 1) is a distinguishing coefficient to adjust the range of the comparison environment, which was selected as 0.5 in this study. Finally, the grey relational grade *γ* was obtained by calculating the average values of all the grey relational coefficients:
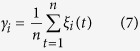
with the values above, the influential degree of the factors on the system could be identified.

### Analytical methods

Color reactions including the Anthrone reaction and the Coomassie brilliant blue reaction were carried out to identify the main components of P-GS408. Nucleic acid was determined qualitatively by a UV–Vis spectrophotometer (Lambda 25, PerkinElmer, USA). Proteins were determined using the Bradford method with bovine albumin (Amresco) as the standard. Total sugar was determined using the phenol-sulfuric acid method with glucose as the standard. For further analysis of monosaccharide composition, the purified P-GS408 was hydrolyzed by 4 M CF_3_COOH for 4 h at 100 °C in a sealed hydrolysis tube. After cooling, the solution was reduced by NaBH_4_ in 0.05 M NaOH at 70 °C. The residual NaBH_4_ was removed by coevaporation with methanol after the solution was hydrolyzed by glacial acetic acid. Finally, the sample was O-acetylated with acetic anhydride and pyridine, and the obtained alditol acetates were determined by a GC-MS (Trace DSQ, Thermo, USA)^20^.

The electric charge of P-GS408 was classified by the addition of cetylpyridium chloride (CPC). The surface morphology of purified P-GS408, kaolin particles and resulted flocs were observed by environmental scanning electron microscope (ESEM) (Quanta 200, FEI, USA) after coated with gold. Functional groups of purified P-GS408 samples were obtained on a KBr disk by FTIR (Nicolet 360, Thermo, USA). Elemental composition of P-GS408 was analyzed using XPS (AXIS UltraDLD, Shimazu, Japan). The zeta potential was measured using a NanoZ zeta potential analyzer (Malvern, UK). Three-dimensional excitation-emission (EEM) fluorescence spectra were obtained using a luminescence spectrometry (LS-55, Perkin-Elmer, USA) the scanning wavelength range was set as: excitation wavelength (Ex) 200–400 nm, emission wavelength (Em) 300–550 nm. The spectrum of distilled water was recorded as the blank. To solve the overlap problem of the fluorescence spectra of complex composition, PARAFAC analysis was employed to separate the actual fluorescence spectra from the EEM fluorescence spectra^31^. The software Matlab 7.0 (MathWorks Inc., USA) was employed for handling the EEM data and PARAFAC modeling.

## Additional Information

**How to cite this article**: Yu, L. *et al*. A novel Fe(III) dependent bioflocculant from *Klebsiella oxytoca* GS-4-08: culture conditions optimization and flocculation mechanism. *Sci. Rep.* **6**, 34980; doi: 10.1038/srep34980 (2016).

## Supplementary Material

Supplementary Information

## Figures and Tables

**Table 1:** CCD and experimental results for three variables in actual factor.

Run	*x*_1_	*x*_2_	*x*_3_	*Y* (%)		*ξ*_1_	*ξ*_2_	*ξ*_3_
				Measure	Predicted			
1	10	3	16	3.9	16.7	0.376	0.3759	0.6694
2	3.3	2.25	32	56.3	46.5	0.5118	0.8545	0.8542
3	5	3	16	4.6	17.6	0.5857	0.3787	0.6783
4	10	1.5	48	74.2	72.3	0.8109	0.4618	1.0000
5	10	1.5	16	17.7	5.5	0.4439	0.7572	0.9197
6	7.5	2.25	32	97.9	99.2	0.4171	0.4172	0.4171
7	5	1.5	48	88.0	86.3	0.3888	0.3888	0.7109
8	7.5	2.25	32	97.6	99.2	0.4185	0.4186	0.4185
9	11.7	2.25	32	40	34.1	0.5229	0.8854	0.8857
10	7.5	0.99	32	26.1	46.4	0.6502	0.9393	0.6502
11	5	3	48	16.34	39.7	0.7355	0.4362	0.396
12	7.5	2.25	58.9	99.4	81.0	0.4096	0.4097	0.7931
13	5	1.5	16	19.5	10.3	0.7897	0.7895	0.9679
14	7.5	2.25	32	99.5	99.2	0.4091	0.4091	0.4091
15	10	3	16	3.4	6.1	0.4528	0.4528	0.9727
16	3.3	2.25	32	9.5	29.7	0.4006	0.4006	0.3664
17	5	3	16	52.5	16.6	0.9443	0.6501	0.9443
						0.5451	0.5544	0.709

**Table 2:** Functional groups analysis of XPS results.

Element	Binding energy (eV)	Corresponding functional groups
C	284.1	C-(C,H) of lipids or amino acid side chains
	285.5	C-(O,N) of alcohol, ether amine, or amide
	287.4	O=C-O of carboxylate, carbonyl, or amide
	289.0	C=O of carboxyl or ester
O	531.5	O (C=O) of carboxylate, carbonyl, or amide
	533.4	C-O of alcohols, hemiacetal, or acetal
N	398.8	amines and amides
	401.4	amino sugars
