# Supplementary material for: A novel Fe(III) dependent bioflocculant from Klebsiella oxytoca GS-4-08: culture conditions optimization and flocculation mechanism
Source: Sci Rep. 2016 Oct 7;6:34980. doi: 10.1038/srep34980 (PMC5054519; doi:10.1038/srep34980)
Supplement: Supplementary Information [file srep34980-s1.pdf]

Supplementary material

A novel Fe(III) dependent bioflocculant from *Klebsiella oxytoca* GS-4-08: culture conditions optimization and flocculation mechanism

Lei Yu[1,*], Qing-wen Tang[1], Yu-jia Zhang[1], Rong-ping Chen[1], Xin Liu[1], Wei-chuan Qiao[1], Wen-wei Li[2], Hong-hua Ruan[1], Xin Song[3,*]

[1] College of Biology and the Environment, Co-Innovation Center for Sustainable Forestry in Southern China, Nanjing Forestry University, Nanjing, 210037 China

[2] CAS Key Laboratory of Urban Pollutant Conversion, Department of Chemistry, University of Science & Technology of China, Hefei, 230026 China

[3] Key Laboratory of Soil Environment and Pollution Remediation, Institute of Soil Science, Chinese Academy of Science, Nanjing 210008, China

*** Corresponding Author:** Lei Yu and Xin Song

Fax: +86 2585427024; E-mail: lyu@njfu.edu.cn; xsong@issas.ac.cn

Table S1 ANOVA analysis

Source	Sum of square	Degree of freedom	Mean square	F-value	*p*-value
Model	19484.83	9	2164.98	4.09	0.0384
Residual	3705.8	7	529.4		
Lack of Fit	3703.74	5	740.75	722.04	0.0014
Pure Error	2.05	2	1.03		
Cor Total	23190.62	16			

R^2 = 0.840

Table S2 Estimated regression coefficients and significance of quadratic model of flocculation efficiency

Parameter	Regression coefficients	Degrees of freedom	Standard error	F-value	Prob > F
Intercept	99.23	1	13.26		
x_1	-3.7	1	6.23	0.35	0.5705
x_2	-8.85	1	6.23	2.02	0.1983
x_3	22.24	1	6.23	12.76	0.0091
x_1x_2	1.01	1	8.13	0.015	0.9051
x_1x_3	-2.28	1	8.13	0.078	0.7878
x_2x_3	-13.46	1	8.13	2.74	0.1421
x_1^2	-20.83	1	6.85	9.24	0.0188
x_2^2	-23.95	1	6.85	12.22	0.0101
x_3^2	-19.69	1	6.85	8.25	0.0239

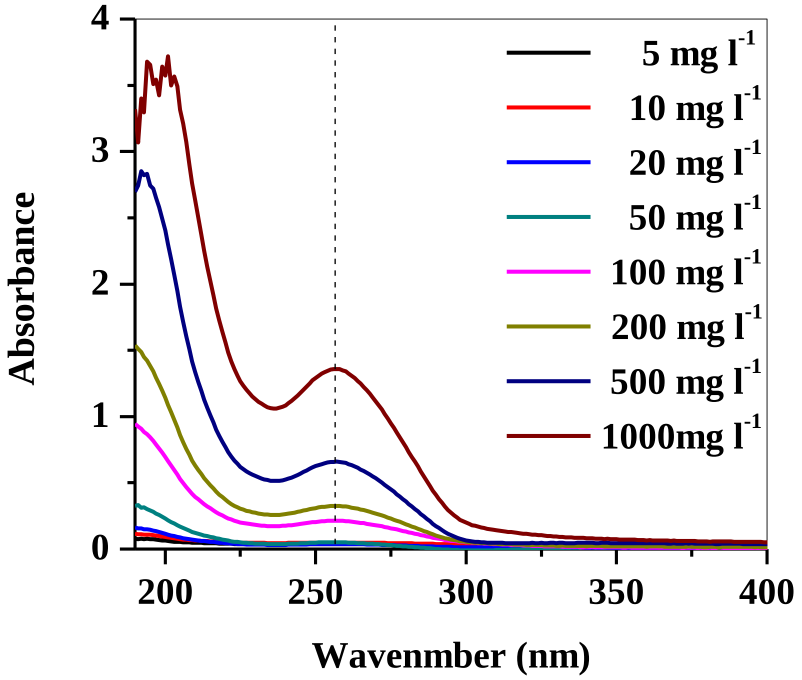

Figure S1. UV-Vis spectrum of different P-GS408 concentrations varied from 5 mg to 1000 mg per liter.

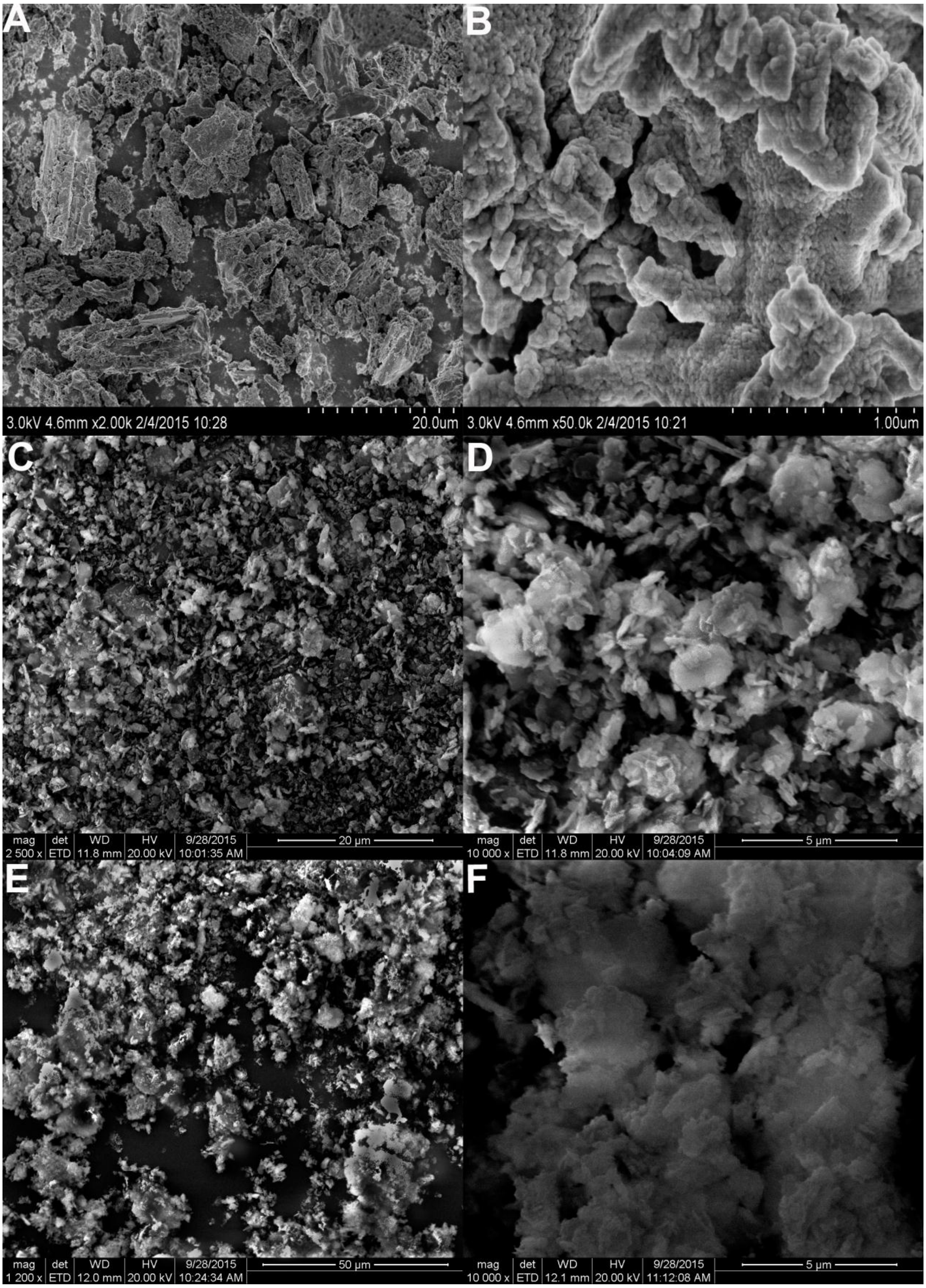

Figure S2. SEM pictures of purified P-GS408 (A) and (B); Kaolin particles (C) and (D); floc (E) and (F)

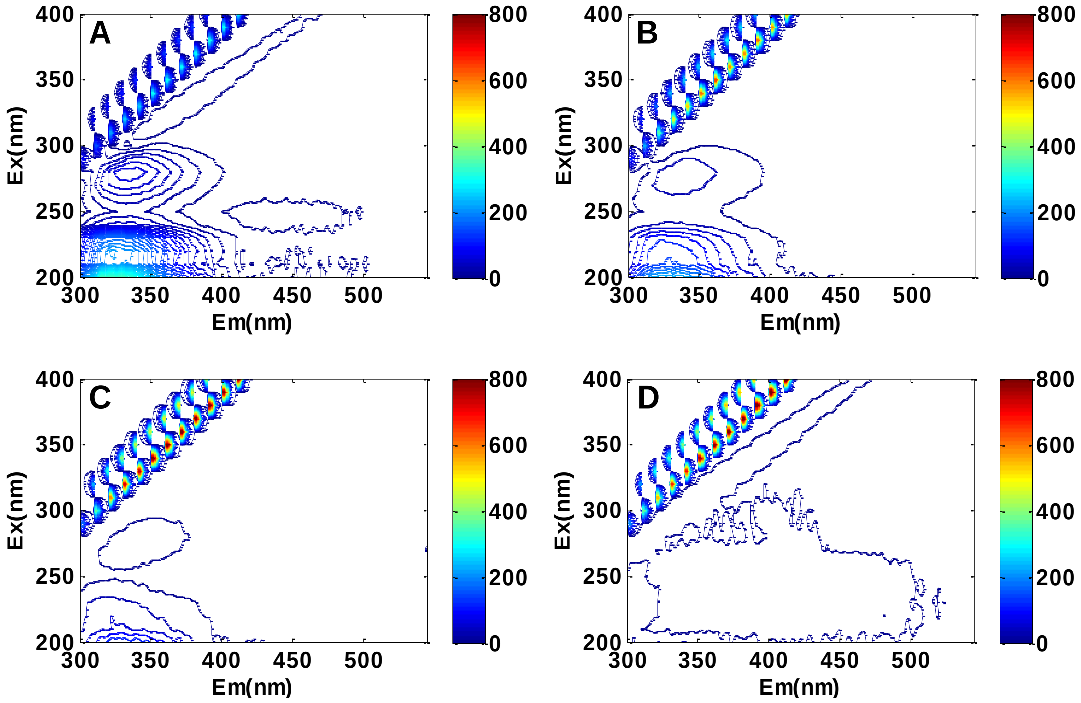

Figure S3. EEM spectra of P-GS408 with different Fe^{3+} concentrations: (A) 0.1 mM; (B) 0.2 mM; (C) 0.4 mM; (D) 1.0 mM.
